# Loss of genes implicated in gastric function during platypus evolution

**DOI:** 10.1186/gb-2008-9-5-r81

**Published:** 2008-05-15

**Authors:** Gonzalo R Ordoñez, LaDeana W Hillier, Wesley C Warren, Frank Grützner, Carlos López-Otín, Xose S Puente

**Affiliations:** 1 Departamento de Bioquímica y Biología Molecular, Facultad de Medicina, Instituto Universitario de Oncología, Universidad de Oviedo, C/Fernando Bongera s/n, 33006 Oviedo, Spain; 2 Genome Sequencing Center, Washington University School of Medicine, Campus Box 8501, 4444 Forest Park Avenue, St. Louis, Missouri 63108, USA; 3 Discipline of Genetics, School of Molecular & Biomedical Science, The University of Adelaide, 5005 South Australia, Adelaide, Australia

## Abstract

**Background:**

The duck-billed platypus (*Ornithorhynchus anatinus*) belongs to the mammalian subclass Prototheria, which diverged from the Theria line early in mammalian evolution. The platypus genome sequence provides a unique opportunity to illuminate some aspects of the biology and evolution of these animals.

**Results:**

We show that several genes implicated in food digestion in the stomach have been deleted or inactivated in platypus. Comparison with other vertebrate genomes revealed that the main genes implicated in the formation and activity of gastric juice have been lost in platypus. These include the aspartyl proteases pepsinogen A and pepsinogens B/C, the hydrochloric acid secretion stimulatory hormone gastrin, and the α subunit of the gastric H^+^/K^+^-ATPase. Other genes implicated in gastric functions, such as the β subunit of the H^+^/K^+^-ATPase and the aspartyl protease cathepsin E, have been inactivated because of the acquisition of loss-of-function mutations. All of these genes are highly conserved in vertebrates, reflecting a unique pattern of evolution in the platypus genome not previously seen in other mammalian genomes.

**Conclusion:**

The observed loss of genes involved in gastric functions might be responsible for the anatomical and physiological differences in gastrointestinal tract between monotremes and other vertebrates, including small size, lack of glands, and high pH of the monotreme stomach. This study contributes to a better understanding of the mechanisms that underlie the evolution of the platypus genome, might extend the less-is-more evolutionary model to monotremes, and provides novel insights into the importance of gene loss events during mammalian evolution.

## Abstract 2

Several genes implicated in food digestion have been deleted or inactivated in platypus. This loss perhaps explains the anatomical and physiological differences in the gastrointestinal tract between monotremes and other vertebrates and provides insights into platypus genome evolution.

## Background

A major goal in the sequencing of different genomes is to identify the genetic changes that are responsible for the physiological differences between these organisms. In this regard, the comparison between human and rodent genomes has identified an expansion in rodents of genes that are implicated in fertilization and sperm maturation, host defense, odor perception, or detoxification [1-3], confirming at the genetic level the physiological differences in these processes between humans and rodents. Additionally, the development of specific biological processes during evolution, for example the production of milk in mammals, has been accompanied by the appearance of novel genes that are implicated in these novel functions, such as casein and α-lactalbumin [4]. Therefore, it appears that the acquisition of novel physiological functions during vertebrate evolution has been driven by the generation of novel genes adapted to these newer functions. However, although gene gains constitute an intuitive mechanism for the development of novel biological functions, gene losses have also been important during evolution, both quantitatively and qualitatively [5-9]. The recent availability of numerous vertebrate genomes has opened the possibility to perform large-scale evolutionary analysis in order to identify differential genes responsible for the specific differences in particular biological processes.

The duck-billed platypus (*Ornithorhynchus anatinus*) represents a valuable resource for unraveling the molecular mechanisms that have been active during mammalian evolution, due both to its phylogenetic position and to the presence of unique biological characteristics [10]. Together with the echidnas, platypus constitutes the Monotremata subclass (prototherians); this is one of the two subclasses into which mammals are divided, together with therians, which are further subdivided into marsupials (metatherians) and placental mammals (eutherians) [11]. The appearance of mammal-specific characteristics such as homeothermy, presence of fur, and mammary glands makes this organism a key element in elucidating the genetic factors that are implicated in the appearance of these biological functions. Nevertheless, since the last mammalian common ancestor, more than 166 million years ago (MYA) [12,13], other characteristics have emerged, such as the presence of venom glands or electroreception, and some vertebrate characteristics have been lost, resulting in the absence of adult teeth or a functional stomach [14,15].

**Figure 1:**
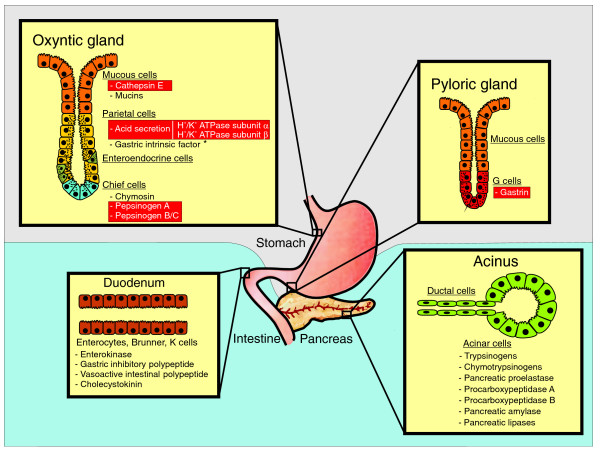
Scheme of the eutherian gastrointestinal system, showing gastric glands and specific cell types. Proteins secreted by each cell type and directly implicated in food digestion are indicated, highlighting in red those proteins that are absent in platypus. *Gastric intrinsic factor is produced by parietal cells in humans but in the pancreas of monotremes and other mammals.

In this work, we show that there has been a selective deletion and inactivation in the platypus genome of several genes that are implicated in the activity of the stomach, including all genes encoding pepsin proteases, which are involved in the initial digestion of proteins in the acidic pH of the stomach, as well as the genes required for the secretion of acid in this organ (Figure 1). The loss and inactivation of these genes provide a molecular basis for understanding the mechanisms that are responsible for the absence in platypus of a functional stomach, and expand our knowledge of the evolution of mammalian genomes.

## Results and discussion

### Loss of pepsin genes in the platypus genome

**Figure 2:**
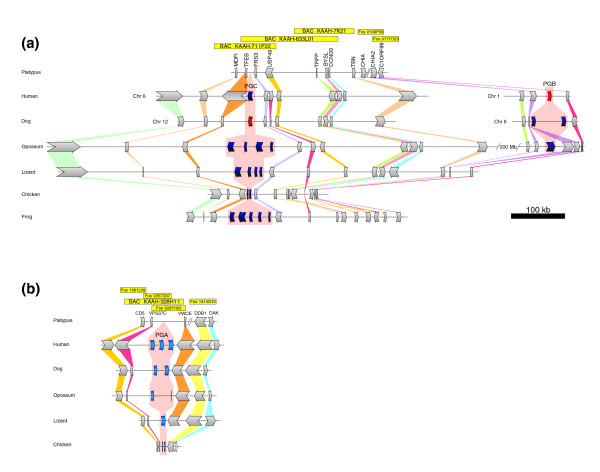
Deletion of pepsinogen-coding genes in the platypus genome. **(a) **Synteny map of the loci containing *PGB *and *PGC *in vertebrates shows a strong conservation of the genes encoding pepsinogen C and its flanking genes, with the exception of platypus, in which *PGC *has specifically been deleted. The figure also shows how the gene encoding pepsinogen B appeared in therians as a result of a duplication of *PGC *to a nearby locus, followed by a translocation. The corresponding region in the platypus genome lacks any pepsinogen-coding gene. Functional pepsinogen genes are colored in blue, whereas pepsinogen pseudogenes are in red. For human and dog, which underwent a translocation of the *PGB *locus, chromosomes are indicated on the left. The genome sequences analyzed are from platypus (*Ornithorhynchus anatinus*), human (*Homo sapiens*), dog (*Canis familiaris*), opossum (*Monodelphis domestica*), lizard (*Anolis carolinensis*), chicken (*Gallus gallus*), and frog (*Xenopus tropicalis*). **(b) **Synteny map of the *PGA *locus in different vertebrate species shows the deletion of this gastric protease gene in the platypus genome. Bacterial artificial chromosomes (BACs) and fosmids used in the study are indicated at the top of each panel. Gene colors and scale are the same as in panel a.

During the initial annotation and characterization of the platypus genome, we noticed the absence of several protease genes in this organism that were present in other mammalian species [2,10]. Most of these lost protease genes encode members of rapidly evolving protease families, including proteases that are implicated in immunological functions, spermatogenesis, or fertilization [2,16]. However, when we performed a further detailed analysis of all of these protease genes lost in platypus, we observed that those encoding three major gastric aspartyl proteases (pepsinogen A, pepsinogen B, and gastricsin/pepsinogen C) were also absent from the platypus genome assembly. These proteases are responsible for the proteolytic cleavage of dietary proteins at the acidic pH of the stomach, and have been highly conserved through evolution, from fish to mammals and birds [17]. The genes encoding these proteases (*PGA*, *PGB*, and *PGC*) are located in different chromosomal loci, whose overall structure has also been well conserved in most vertebrate genomes, including platypus (Figure 2). Therefore, it appeared unlikely that their absence in platypus could be due to the incompleteness of the genome assembly in a specific chromosomal region. Moreover, analysis of more than 2 million trace sequences not present in the assembly and expressed sequence tag (EST) sequences from different platypus tissues [10] also failed to reveal the existence of any of these pepsinogen genes, reinforcing the hypothesis that they had been specifically deleted in the genome of this mammal.

To investigate this possibility further, we first compared the genomic organization of these three aspartyl protease genes - *PGA*, *PGB *and *PGC *- in the genomes of human, dog, opossum, chicken, lizard, and frog [18-21]. It is well established that the genes encoding pepsinogens have undergone several expansions during vertebrate evolution, leading to the presence of at least three to six distinct functional members in the genomes of these organisms (Figure 2a). Additionally, a duplication event in *PGC *in the therian lineage has resulted in the formation of *PGB*, which appears to be functional in opossum and dog, and in the latter has probably replaced the function of *PGC*, which has been inactivated by pseudogenization. The loci containing these pepsinogen genes have been highly preserved through evolution, and their flanking genes are also perfectly conserved in both order and nucleotide sequence in vertebrate genomes (Figure 2a).

Analysis of platypus bacterial artificial chromosomes (BACs) and/or fosmids corresponding to these regions revealed that the genes flanking the pepsinogen genes in other species are conserved and map to the corresponding syntenic region of the platypus genome (Figure 2). However, a DNA probe corresponding to murine pepsinogen A failed to hybridize with the analyzed platypus BACs or fosmids spanning the regions of interest (see Additional data file 1). Moreover, complete sequencing of the platypus genomic regions flanked by *TFEB *and *FRS3 *as well as by *C1orf88 *and *CHIA2 *failed to detect any genes encoding pepsinogen C or pepsinogen B, respectively. Additionally, and in order to test the possibility that pepsinogen genes have been transposed to other loci during platypus evolution, a Southern blot analysis with the same probe was performed using total genomic DNA. This analysis resulted in the absence of hybridization when genomic DNA from platypus and one echidna species (*Tachyglossus aculeatus*) were used, whereas the same probe readily detected two hybridization bands in more evolutionary distant species such as lizard (*Podarcis hispanica*) and chicken (data not shown).

Together, these data indicate that the genes encoding these gastric proteases have been specifically deleted in the genome of monotremes, probably resulting in important differences in the digestion of dietary proteins in these species when compared with other vertebrates.

### Loss or inactivation of platypus genes implicated in stomach acid secretion

Pepsinogens are synthesized by chief cells in the oxyntic glands of the stomach as inactive precursors that become activated when they are exposed to the low pH of the gastric fluid [22]. The secretion of hydrochloric acid is stimulated by the gastric hormone gastrin, which is released by enteroendocrine G cells that are present in pyloric glands in response to amino acids and digested proteins. To try to extend the above findings on the absence of pepsinogen genes in platypus, we next evaluated the possibility that the gene encoding gastrin (*GAST*) could also be absent from the platypus genome.

**Figure 3:**
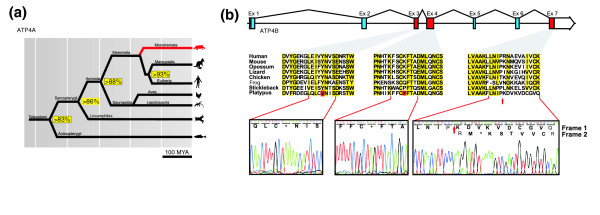
Absence of a functional gastric acid secreting H^+^/K^+^-ATPase in monotremes. **(a) **Phylogenetic tree showing the distribution of a functional α subunit of the H^+^/K^+^-ATPase gene (*ATP4A*) in vertebrates, indicating in red the absence of this gene in platypus. The percentage of identities at the protein level of ATP4A from human (*Homo sapiens*), dog (*Canis familiaris*), opossum (*Monodelphis domestica*), lizard (*Anolis carolinensis*), chicken (*Gallus gallus*), and frog (*Xenopus tropicalis*) is shown in yellow boxes. **(b) **Gene structure of *ATP4B *and amino acid sequence alignment of the indicated exons with ATP4B from different vertebrate species, including the teleost fish stickleback (*Gasterosteus aculeatus*). Electropherograms and sequence translation of platypus *ATP4B *exons 3, 4, and 7 showing the presence of premature stop codons and a frameshift (red arrow). MYA, million years ago.

After comparative genomic analysis following the same strategy as in the case of pepsinogen genes, we failed to detect any evidence of the presence of *GAST *in platypus (see Additional data file 1), which suggests that acid secretion might also be impaired in this species. Consistent with this observation, parallel genomic analysis also showed that the α subunit of the H^+^/K^+^-ATPase (*ATP4A*), which is responsible for the acidification of the stomach content by parietal cells, has also been deleted from the platypus genome. This gene, which is present from fish to amniotes, has been highly conserved through evolution but is absent from the platypus genome assembly (Figure 3a). Also similar to the case of pepsinogen genes, the *ATP4A*-flanking genes (*TMEM147 *and *KIAA0841*), which are present in fish, therians, and chicken, were readily identified in platypus. Thus, analysis of a fosmid clone corresponding to this region with a probe for the most proximal gene (*TMEM147*) resulted in detection of a specific hybridization band in platypus (see Additional data file 1). However, no hybridization bands could be detected in platypus fosmid KAAG-0404B19, or total genomic DNA from platypus and *T. aculeatus *when using a human derived *ATP4A *probe, which otherwise recognized specific bands in mouse, chicken, and lizard (Additional data file 1 and data not shown). These results extend the above findings on gastric protease genes and demonstrate that other genes involved in the digestive activity of gastric juice have also been selectively deleted from the genomes of monotremes.

We next examined the possibility that mechanisms distinct from those involving the specific deletion of gastric genes could also contribute to the apparent loss in platypus of evolutionarily conserved digestive functions. This analysis led us to conclude that two well known gastric genes - namely *CTSE *and *ATP4B *[23-25], which encode the aspartyl protease cathepsin E and the β subunit of the H^+^/K^+^-ATPase, respectively - have been inactivated by pseudogenization. Thus, we first observed that the platypus genome contains sequences with high similarity to both gastric genes in the corresponding syntenic regions, suggesting that *CTSE *and *ATP4B *could indeed be functional genes in platypus. However, further detailed analysis of their nucleotide sequence revealed that *CTSE *is nonfunctional in this species due both to the presence of a premature stop codon in exon 7 (Lys295Ter) and to the loss of six of its nine exons. Similarly, the gene encoding ATP4B has been pseudogenized in platypus because of the presence of premature stop codons in exons 3 and 4 (Tyr98Ter and Lys153Ter), as well as a frameshift in exon 7 (Figure 3b). This observation, together with the loss of *ATP4A *in platypus, confirms the absence of a functional H^+^/K^+^-ATPase in this vertebrate and provides at least part of the explanation for the lack of acid secretion in the platypus stomach; this is a characteristic feature of monotremes, whose gastric juice is above pH 6 [14].

### Loss of gastric genes during platypus evolution

The mammalian stomach is lined with a glandular epithelium that contains four major cell types [26]: mucous, parietal, chief, and enteroendocrine cells. The data presented above show that the genes encoding different products of these four major cell types of the gastric glandular epithelium have been selectively deleted or inactivated during monotreme evolution (Figure 1 and Table 1). Although the genes encoding proteases have been shown to be subjected to processes of gene gain/loss events in both vertebrate and invertebrate genomes [5,16,27], we have determined that these gene loss events observed in platypus gastric genes do not represent a general process affecting all proteins that are involved in food digestion, because analysis of genes implicated in gastrointestinal functions revealed that those encoding proteases and hormones expressed in the intestine or exocrine pancreas from eutherians are perfectly conserved in platypus (Figure 1). It therefore appears that there has been a selective loss of platypus genes responsible for the biological activity of gastric juice.

**Table 1:** Summary of genes implicated in gastric function in platypus

Protein	Gene	Status in platypus genome	Confirmatory evidence
ATPase, H^+^/K^+ ^exchanging, α polypeptide	*ATP4A*	Absent	Southern blot
ATPase, H^+^/K^+ ^exchanging, β polypeptide	*ATP4B*	Pseudogene	PCR/direct sequencing
Cathepsin E	*CTSE*	Pseudogene	PCR/direct sequencing
Gastrin	*GAST*	Absent	Southern blot
Neurogenin 3	*NGN3*	Absent	Southern blot
Pepsin A	*PGA*	Absent	Southern blot/sequencing
Pepsin C	*PGC*	Absent	Southern blot/sequencing
Gastric intrinsic factor	*GIF*	Present (expression pancreatic)	RT-PCR
Chymosin	*CYMP*	Present (expression not detected)	Sequencing/RT-PCR

RT,-PCR, reverse transcription polymerase chain reaction.

To address this question further, we next performed a detailed search for the putative occurrence in the platypus genome of functional genes encoding proteins secreted by gastric glands. This search led us to the identification of two genes with interesting characteristics in this regard. The gene encoding gastric intrinsic factor (*GIF*), which is necessary for the absorption of vitamin B_12_, is perfectly conserved in platypus. This protein is secreted by chief or parietal cells in most eutherians, but it is mainly produced by pancreatic cells in dogs as well as in opossum, in which no gastric expression can be detected [28,29]. It is therefore likely that the expression of this gene was pancreatic before the prototherian-therian split, and the intrinsic factor might still be secreted by the pancreas in platypus, where it can exert its physiological function.

To investigate this possibility, we conducted RT-PCR analysis using specific primers for *GIF *and RNA from different tissues from either platypus or echidna (*T. aculeatus*). This allowed us to find that *GIF *expression can be detected in pancreas, and lower expression could be also detected in liver as well as in echidna brain, whereas no expression was detected in muscle or brain from platypus (see Additional data file 2). Therefore, these findings indicate that, similar to the case of marsupials, the *GIF *gene is also expressed by the pancreas in monotremes. A similar situation could occur in the case of chymosin, an aspartyl protease that participates in milk clotting by limited proteolysis of κ casein [30]. Chymosin is present in chicken and in most mammalian species, although it has been inactivated by pseudogenization in humans and other primates [2,31]. Our genomic analysis also detected a gene containing a complete open reading frame that might constitute a functional chymosin gene in the platypus genome. This finding, together with the absence of soluble pepsins and cathepsin E in platypus, suggests that chymosin might be the only aspartyl protease with ability to contribute to food digestion in the stomach of platypus. Nevertheless, it is very unlikely that chymosin could compensate for the lack of pepsin activity in platypus stomach because of its much lower proteolytic activity when compared with that of pepsins [30]. Additionally, the high pH of platypus stomach might prevent the zymogen activation and proteolytic activity of this peptidase. Finally, it is possible that, similar to the case of the intrinsic factor, platypus chymosin might be also produced by other tissues. In this regard, we have been unable to detect the expression of this gene in any of the tissues analyzed above (data not shown), although its putative participation in the digestion of dietary proteins should be further characterized.

The loss of stomach function in prototherians is unique among vertebrates, because this organ has been functional for more than 400 million years, from fish to therians and birds, and it has been adapted to specific dietary habits, resulting in the formation of multiple chambers in birds and ruminants [32]. In contrast, the stomach of platypus is completely aglandular and has been reduced to a simple dilatation of the lower esophagus [14,15]. It is remarkable that some fish species such as zebrafish (*Danio rerio*) and pufferfish (*Takifugu rubripes*) have also lost their gastric glands during evolution, although this fact has not apparently resulted in the loss of so many gastric genes in these teleosts as in platypus [33,34]. On the other hand, the small stomach, high pH of gastric fluid, and lack of gastric glands in echidna, together with the finding that some of the gastric genes lost in platypus are also absent in *T. aculeatus*, suggest that the loss of the stomach function and gastric genes in monotremes occurred before the platypus-echidna split, more than 21 MYA [10]. However, it is difficult to determine whether the loss of gastric genes in platypus has conferred a selective advantage during evolution, or whether they have been lost as a result of a relaxed constraint due to additional changes in this species.

In this regard, it is possible that the loss of gastric genes in monotremes might have conferred a selective advantage to this population against parasites or pathogens that rely on the presence of an acidic pH in the stomach for their infection or propagation, or the use of cell surface proteins such as ATP4A, ATP4B, or CTSE as receptors for the infection. Should this be the case, then this would represent a clear example of the 'less-is-more' hypothesis [35,36], which postulates that the loss of a gene might confer a selective advantage under specific conditions. Nevertheless, in the absence of additional data, it cannot be ruled out that additional changes in the digestive system of monotremes made irrelevant the function of the genes described in this work, and they were subjected to the accumulation of deleterious mutations because of a relaxed constraint. However, an interesting question at this point is whether additional strategies have been adopted by platypus to accomplish efficient protein digestion in the absence of a number of gastric enzymes. Changes in dietary habits, such as feeding on insect larvae, which are easily digested; the presence of specific anatomical structures, such as grinding plates or cheek-pouches, which allow food trituration and storage; and the putative occurrence of a characteristic gastrointestinal flora in platypus might constitute mechanisms by which this species has overcome the loss of a functional stomach.

Another question raised by this comparative genome analysis is whether the loss of all of the above discussed genes is cause or consequence of this particular platypus gastric phenotype. Deletion of the gene encoding gastrin might have contributed to this process, because mice deficient in gastrin exhibit an atrophy of the oxyntic mucosa, with a reduced number of parietal and enteroendocrine cells, achlorhydria, and decreased mucosa thickness [37-39]. Additionally, inactivation of *ATP4B *has been shown to produce a significant decrease in pepsin-producing chief cells and alterations in the structure of parietal cells [25]. Moreover, loss of *PGA *might also contribute to the gastric atrophy observed in platypus, because this protease was recently shown to be required for the processing and activation of the morphogen sonic hedgehog (Shh) in the stomach [40]. Therefore, deletion or inactivation of gastrin, the acid-secreting ATPase, and pepsinogen A could have contributed to a substantial reduction in the formation of gastric glands in monotremes. Nevertheless, we cannot discard the possibility that the stomach function was lost by some other unrelated mechanism, and - in the absence of a selective pressure to maintain the genes encoding proteins implicated in the gastric function - these genes were lost by pseudogenization and/or deletion events. However, the exclusive absence of these genes cannot explain the significant reduction in size observed in the stomach of platypus, suggesting that other factors might be responsible for this characteristic feature.

To evaluate this possibility, we first selected a series of genes previously described to influence stomach size in mice and examined its putative presence and sequence conservation in the platypus genome (Additional data file 3). This analysis allowed us to determine that the gene encoding neurogenin-3 has been lost in platypus (Additional data file 1 and Table 1).

Neurogenin-3 is a transcription factor whose activity is required for the specification of gastric epithelial cell identity, and deficiency of this factor results in considerably smaller stomachs and absence of gastrin-secreting G cells, somatostatin-secreting D cells and glucagon-secreting A cells [41]. Therefore, it is tempting to speculate that neurogenin-3 could be a candidate gene to explain, at least in part, the morphological differences between platypus stomach and that of other vertebrates. Nevertheless, further studies of the role of neurogenin-3 in different species will be required to ascribe a role to this transcription factor in defining structural or functional differences in stomach during mammalian evolution.

### Mechanisms involved in the loss of gastric genes in platypus

**Figure 4:**
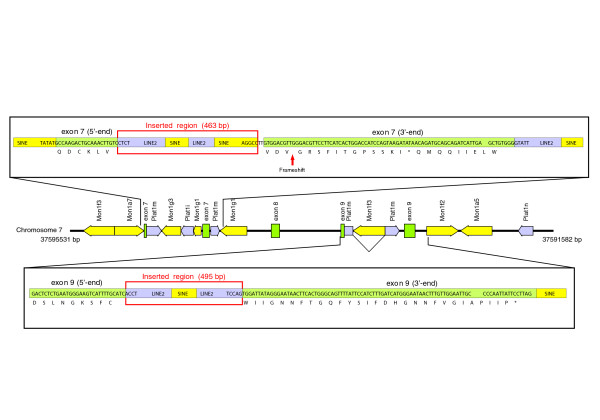
Inactivation of *CTSE *gene by insertion of interspersed elements. Genetic map of the *CTSE *locus in the platypus genome showing the disruption of exons 7 and 9 by interspersed elements. Top and bottom panels show a more detailed view of exons 7 and 9, respectively, indicating the nucleotide sequence of exons and the disrupting long interspersed element (LINE)2 and short interspersed element (SINE) elements. bp, base pairs.

Finally, in this work we have also examined putative mechanisms responsible for the loss of gastric genes in the platypus genome. A first possibility in this regard should be the occurrence of directed gene losses specifically occurring in platypus and the two extant echidna species *Zaglossus *and *Tachyglossus*. As a first step in this analysis, and based on recent studies of specific gene losses during hominoid evolution [42], we examined the hypothesis that gastric genes were independently deleted in platypus by nonallelic homologous recombination or by insertion of repetitive sequences. Consistent with this possibility, and in agreement with the increased activity of interspersed elements in the platypus genome [10,43], we have found that the *CTSE *gene has been disrupted in platypus by the insertion of long interspersed elements (LINEs) and short interspersed elements (SINEs) in exons 7 and 9, disrupting the protein coding region (Figure 4). Interestingly, exon 9 was disrupted by the insertion of a LINE2 Plat1m element, which was further disrupted by the insertion of a SINE Mon1f3 element (Figure 4). In this regard, analysis of different interspersed elements in the platypus genome has revealed that the main period of activity of Mon1f3 elements was between 88 and 159 MYA [10], indicating that pseudogenization of *CTSE *might have occurred within this period, and suggesting that the inactivation of gastric genes in monotremes started at least 88 MYA. Furthermore, the high abundance of repetitive elements in the *CTSE *region (more than 3.8 interspersed elements per kilobase as compared with 2 for the genome average [10]) might have contributed to the deletion of six out of the nine exons of *CTSE *by nonallelic homologous recombination between these repetitive elements. The variable density of interspersed elements in the regions examined in this study raises the possibility that similar mechanisms to that observed in *CTSE *might have been responsible for the complete deletion of other gastric genes, although the participation of other mechanisms in this process cannot be ruled out.

## Conclusion

In summary, detailed analysis of the platypus genome sequence has allowed us to demonstrate that a number of genes that are implicated in food digestion in the stomach have specifically been deleted or inactivated in this species, as well as in echidna. It is remarkable that the results presented here may constitute an exceptional example of the less-is-more evolutionary model [35,36], both for the number of genes involved as well as for the physiological consequences derived from these genetic losses. In fact, the loss of the gastric genes reported in this study appears to be responsible for the specific characteristics of the platypus gastrointestinal system, although it cannot be ruled out that the loss of the stomach by other unrelated events might have resulted in the neutral evolution of these genes. The gastric genes lost in the platypus genome include those encoding the aspartyl proteases pepsinogen A, pepsinogens B/C and cathepsin E, the hydrochloric acid secretion stimulatory hormone gastrin, and both subunits of the gastric H^+^/K^+^-ATPase. Likewise, genes encoding proteins implicated in stomach development, such as the neurogenin-3 transcription factor, are also absent in the platypus genome. All of these genes have been highly conserved in vertebrates for more than 400 million years, reflecting a unique pattern of evolution in the platypus genome when compared with other mammalian genomes. On the basis of these findings, we propose that loss of genes involved in gastric functions might be responsible for the remarkable anatomical and physiological differences of the gastrointestinal tract between monotremes and other vertebrates, and underscores the importance of gene loss for mammalian evolution.

## Materials and methods

### Bioinformatic analysis

The identification of protease-coding genes in the platypus genome was carried out as previously described [27], using a 6X assembly (version 5.0) generated with the PCAP assembly program, with an estimated coverage of 90% to 93% [10]. Briefly, protein sequences corresponding to human proteases were searched in the platypus assembly using the TBLASTN algorithm with an expected threshold of 10. In most cases this was sufficient to identify individual contigs containing exons with high sequence identity to the queried protease, which were further analyzed to obtain the full-length coding sequence. In those cases in which no clear ortholog was found in the platypus genome assembly, the following procedure was used. First, the traces and the EST sequences were analyzed using BLASTN and TBLASTN, increasing the expected threshold up to 1,000, which was sufficient to detect the orthologous genes in the assembly and traces of more evolutionary distant vertebrates such as lizard, chicken, or frog. Second, to exclude the possibility that these results arose simply because that the human gene was too divergent from the platypus one, the query sequence was replaced by the corresponding ortholog in mouse, dog, opossum, chicken, lizard, frog, or fish (when available), and the search was performed in the platypus assembly, traces, and ESTs using BLASTN and TBLASTN. Third, if the previous strategies failed, then the 5'- and 3'-flanking genes in other vertebrate genomes were used as query to identify platypus contigs corresponding to the locus in which the candidate gene was supposed to lie. These contigs were then searched with the TBLASTN algorithm with increasing expected threshold to identify potential exons of the gene or pseudogene, and the contigs were analyzed for the presence of large gaps. When large gaps were found, BACs and/or fosmids corresponding to those regions were obtained and analyzed by Southern blot and/or sequencing.

### Southern blot and sequencing

Platypus BACs were obtained from Children's Hospital Oakland Research Institute, and fosmids and genomic DNA were provided by the platypus genome sequencing project [10]. DNA was digested with the indicated enzymes, separated in a 0.7% agarose gel, and transferred to a nylon membrane. Southern blot hybridization was performed using specific oligonucleotides corresponding to platypus genes present in the assembly (Additional data file 4) or using human or mouse-derived cDNA probes for ATP4A (corresponding to nucleotides 1,899 to 2,503 of sequence NM_000704), PGA (corresponding to nucleotides 867 to 1,259 of sequence NM_021453), and NGN3 (corresponding to nucleotides 387 to 593 of sequence NM_020999). DNA probes were PCR-amplified using Taq Platinum (Invitrogen, Carlsbad, CA) and purified. All PCRs were performed in a Veriti 96-well thermal cycler (Applied Biosystems, Foster City, CA) for 35 cycles of denaturation (95°C for 15 seconds), annealing (60°C for 15 seconds), and extension (72°C for 30 seconds). Double-stranded DNA probes were radiolabeled with [α-^32^P]dCTP (3,000 Ci/mmol) from GE Healthcare (Uppsala, Sweden), using a commercial random priming kit purchased from the same company. When specific oligonucleotides were used for hybridization, they were labeled with [γ-^32^P]ATP (3,000 Ci/mmol) from GE Healthcare using T4 Polynucleotide Kinase (USB, Cleveland, OH). Hybridization was performed at 42°C or 60°C for oligonucleotides or cDNA probes, respectively, using a Rapid-Hyb hybridization solution (GE Healthcare). Additionally, the regions corresponding to the *PGC *and *PGB *loci in platypus were cloned from the indicated BACs and fosmids, and subjected to direct sequencing using the kit DR terminator *Taq*FS and the automatic DNA sequencer ABI-PRISM 310 (Applied Biosystems), with specific oligonucleotides as primers. Mutations in gastric genes were confirmed by amplification of the corresponding exons with specific primers (Additional data file 4) using platypus genomic DNA as template, and the amplified product was subjected to nucleotide sequencing.

### Analysis of GIF expression in platypus and echidna tissues

Total RNA from platypus and echidna (*T. aculeatus*) tissues was reverse-transcribed using oligo-dT and the RNA-PCR Core kit from Perkin Elmer Life Sciences (Foster City, CA) and subjected to PCR amplification using specific primers for *GIF *(5'-TGGCTCTGACCTGTATGTACA and 5'-GGTTTTGCCTTTCAGG GAAGG) and *GAPDH *(5'-AAGGCTGTGGGCAAGGTCAT and 5'-CTGTTGAAGTCACAGGAGAC).

## Abbreviations

BAC, bacterial artificial chromosome; EST, expressed sequence tag; LINE, long interspersed element; MYA, million years ago; RT-PCR, reverse transcription polymerase chain reaction; SINE, short interspersed element.

## Authors' contributions

GRO, CLO, and XSP conceived of the study, carried out the data analysis and interpretation, and contributed to the writing of the manuscript. LWH and WCW performed the analysis of BAC and Fosmid ends, and provided individual clones for the indicated loci. FG provided platypus and echidna samples. All authors read and approved the final manuscript.

## Additional data files

The following additional data files are available. Additional data file 1 is a figure showing the following: Southern blot analysis of platypus fosmids KAAG-0287H03, KAAG-0109P06, and BAC KAAG-711F22; synteny map of the gastrin locus in the indicated species; synteny map of the neurogenin-3 locus in the indicated species; synteny map of the *ATP4A *locus in different vertebrates and platypus fosmid KAAG-0404B19 corresponding to this region. Additional data file 2 is a figure showing the analysis of GIF expression in platypus and echidna tissues. Additional data file 3 is a table listing genes implicated in stomach size and development and their status in the platypus genome. Additional data file 4 is a table listing the oligonucleotides used for amplification, sequencing, and hybridization of the indicated platypus genes.

## Supplementary Material

Additional data file 1 Presented is a figure. (A) Southern blot analysis of platypus fosmids KAAG-0287H03, KAAG-0109P06, and BAC KAAG-711F22, corresponding to the *PGA*, *PGB*, and *PGC *loci with a murine probe for pepsin (*PGA5*), which failed to hybridize with the indicated platypus clones, whereas specific probes for upstream and downstream genes showed strong hybridization signals. Molecular weight markers are indicated on the left. (B) Synteny map of the gastrin locus in the indicated species. (C) Synteny map of the neurogenin-3 locus in the indicated species showing the position of platypus BAC KAAG-414H19. Southern blot analysis of this BAC resulted in the hybridization with a specific probe for the proximal gene *C1ORF35*, but failed to hybridize with a human-derived probe for neurogenin-3, whereas this probe recognized specific bands in chicken and lizard (*Podarcis hispanica*) genomic DNA. (D) Synteny map of the *ATP4A *locus in different vertebrates and platypus fosmid KAAG-0404B19 corresponding to this region. Southern blot analysis with a specific probe for *TMEM147 *revealed the presence of this gene in fosmid KAAH-0404B19. Hybridization with a human probe for *ATP4A *corresponding to exons 13 to 16 failed to hybridize with platypus fosmid KAAH-0404B19. Click here for file

Additional data file 2 Presented is a figure showing the analysis of GIF expression in platypus and echidna tissues. Total RNA from platypus and echidna (*T. aculeatus*) tissues was subjected to RT-PCR using specific primers for *GIF *and *GAPDH *as control. The amplification products were separated in a 3% agarose gel, showing the highest expression of *GIF *in echidna pancreas, as well as in liver from platypus an echidna, whereas no expression could be detected in platypus brain or muscle. The identity of echidna *GIF *was confirmed by direct nucleotide sequencing of the amplified product. Click here for file

Additional data file 3 Presented is a table listing genes implicated in stomach size and development and their status in the platypus genome. Click here for file

Additional data file 4 Presented is a table listing the oligonucleotides used for amplification, sequencing and hybridization of the indicated platypus genes. Click here for file
