# Supplementary material for: Loss of genes implicated in gastric function during platypus evolution
Source: Genome Biol. 2008 May 15;9(5):R81. doi: 10.1186/gb-2008-9-5-r81 (PMC2441467; doi:10.1186/gb-2008-9-5-r81)
Supplement: Additional data file 3 — Presented is a table listing genes implicated in stomach size and development and their status in the platypus genome. [file gb-2008-9-5-r81-S3.doc]

**Additional data File 3.** List of genes implicated in stomach size and development and their status in the platypus genome.

| **Gene** | **KO Phenotype/Function** | **Platypus** | **References** |
| --- | --- | --- | --- |
| *BAPX1/NKX3.2* | Reduced size of forestomach and abnormal morphology of the stomach | Present | [44] |
| *CDX2/CDX3* | Critical for hindgut development | Present | [45] |
| *FOXA1* | Influence gene expression in endodermally derived tissues including stomach | Present | [46] |
| *FOXL1* | Gastric acid secretion suppressed, pyloric gland cells have reduced levels of pepsinogen | Present | [47] |
| *GATA4* | Lack ventral foregut endoderm. Required for proper epithelial-mesenchymal signaling in the developing stomach | Present | [48] |
| *HES1* | Smaller stomach and structural abnormalities | Present | [49] |
| *HNF3B/FOXA2* | Influence gene expression in endodermally derived tissues including stomach | Present | [46] |
| *KEAP1* | Smaller stomach and hyperkeratosis of the esophagus and forestomach | Present | [50] |
| ***NGN3*** | Small stomach, disorganized and increased thickness of gastric mucosa | **Absent** | [41] |
| *RUNX3* | Abnormal gastric parietal cell morphology | Present | [51] |
| *SHH* | Smaller gastrointestinal tract, intestinal transformation of the stomach epithelium | Present | [52] |
| *SOX11* | Smaller stomach and other abnormalities in different tissues | Present | [53] |
| *SOX17* | Growth and morphogenesis of mid- and hindgut severely affected. extensive apoptosis in foregut endoderm | Present | [54] |
| *TNS3* | Smaller gastrointestinal system, including stomach | Present | [55] |
