# Supplementary material for: Loss of genes implicated in gastric function during platypus evolution
Source: Genome Biol. 2008 May 15;9(5):R81. doi: 10.1186/gb-2008-9-5-r81 (PMC2441467; doi:10.1186/gb-2008-9-5-r81)
Supplement: Additional data file 4 — Presented is a table listing the oligonucleotides used for amplification, sequencing and hybridization of the indicated platypus genes. [file gb-2008-9-5-r81-S4.doc]

**Additional Data File 4.** List of oligonucleotides used in this study

| **Primer Name** | **Sequence** | **Purpose** |
| --- | --- | --- |
| **Sequencing** |  |  |
| ATP4B_E3Fwd | AGAGGGACAGCGAGAAGGTT | Exon 3 ATP4B Sequencing |
| ATP4B_E3Rev | GCCCCATTGCTTTAGGATTT | Exon 3 ATP4B Sequencing |
| ATP4B_E4Fwd | TATCCACTCCCGGCACTTAG | Exon 4 ATP4B Sequencing |
| ATP4B_E4Rev | GCCTCCCTACATAGGGACA | Exon 4 ATP4B Sequencing |
| ATP4Bbis_E4Fwd | TCCCTTCTCTGTGCCTCAGT | Exon 4 ATP4B Sequencing |
| ATP4Bbis_E4Rev | ATTTTAAGGAGTCCGAGGCCA | Exon 4 ATP4B Sequencing |
| ATP4B_E7Fwd | GAATTGCAACGACCAGTCC | Exon 7 ATP4B Sequencing |
| ATP4B_E7Rev | AGGGGTCGTGGGGATTGT | Exon 7 ATP4B Sequencing |
| CTSE_E7Fwd | TGCCTCAGTCCATCAATCAA | Exon 7 CTSE Sequencing |
| CTSE_E7Rev | AGGACAAGTTTGCAGTCTTGG | Exon 7 CTSE Sequencing |
| CTSE_E9Fwd1 | GTCCTTCGGTTTGAACGTGA | Exon 9 CTSE Sequencing |
| CTSE_E9Fwd2 | CCTTCTGGTTCCTTTGAGCTT | Exon 9 CTSE Sequencing |
| CTSE_E9Rev1 | GGGTGAGATGGGAGTGAAAA | Exon 9 CTSE Sequencing |
| CTSE_E9Rev2 | TCCGAAGAGCCTTAGCAGTC | Exon 9 CTSE Sequencing |
| CYMP Fwd | AGGGGCAACTTGGAAAGG | Chymosin amplification and sequencing |
| CYMP Rev | AGAAAAAGCCCTAGCCAACC | Chymosin amplification and sequencing |
| CYMP Int | GGGCCTGTCTCTCTTTTCAG | Chymosin amplification and sequencing |
| CYMP Int Rev | TCATCTTTCCCTTGAGGGTG | Chymosin amplification and sequencing |
| **Southern Blot** |  |  |
| PGC_1 | AATGCAGGGGAGTAACATCG | Southern Blot |
| VPS37C_1 | AGCCTTGGGCTTCCTCATCA | Southern Blot |
| TFEB_E1 | AGCTGCATGCGCAGGCCAAT | Southern Blot |
| VWCE_1 | GGAAGGAAGGTGATGAATGG | Southern Blot |
| TMEM147Fwd | CCTTGTAGGCGATGAGGAAG | Southern Blot |
| MGC10433 | ATAGAGAGCAGCTCGGGGAT | Southern Blot |
| NGN3Fwd | CTGAGCAAGCAGCGACGGA | Human NGN3 probe amplification |
| NGN3Rev | GCTGTGGTCCGCTATGCGC | Human NGN3 probe amplification |
| PEPF Fwd | GCATCTCCATGAATGGGGAGG | Mouse PGA5 probe amplification |
| PEPF Rev | CAGCAGGAGCCAGACCAATCC | Mouse PGA5 probe amplification |
| ATP4A Fwd | GTGATCATGGTAACGGGTGAC | Human ATP4A probe amplification |
| ATP4A Rev | GTGCAGAGTTCGATGAAGAGG | Human ATP4A probe amplification |
| **RT-PCR** |  |  |
| GIF Fwd | TGGCTCTGACCTGTATGTACA | Gastric Intrinsic Factor RT-PCR |
| GIF Rev | GGTTTTGCCTTTCAGGGAAGG | Gastric Intrinsic Factor RT-PCR |
| CYMP Fwd | CGGCTCCTCTGACTTCTGGGT | Chymosin RT-PCR |
| CYMP Rev | CACCCAGCTCTTGGGTGCTCA | Chymosin RT-PCR |
| GAPDH Fwd | AAGGCTGTGGGCAAGGTCAT | GAPDH RT-PCR |
| GAPDH Rev | CTGTTGAAGTCACAGGAGAC | GAPDH RT-PCR |
